# Supplementary material for: Sponsorship Bias in Clinical Trials in the Dental Application of Probiotics: A Meta-Epidemiological Study
Source: Nutrients. 2022 Aug 19;14(16):3409. doi: 10.3390/nu14163409 (PMC9413900; doi:10.3390/nu14163409)
Supplement: Supplementary file 1 [file nutrients-14-03409-s001.zip › nutrients-1851312-supplementary.pdf]

Supplementary materials

Table S1. Search strategy.

**Ovid MEDLINE(R) and Epub Ahead of Print, In-Process, In-Data-Review & Other Non-Indexed Citations and Daily <1946 to April 07, 2022>**	1 (systematic review* or meta-analys*).mp. [mp=title, abstract, original title, name of substance word, subject heading word, floating sub-heading word, keyword heading word, organism supplementary concept word, protocol supplementary concept word, rare disease supplementary concept word, unique identifier, synonyms] 397176<br>2 (dent* or tooth or teeth or orthodon* or oral surg* or endodon* or periodon* or prosthodon* or pedodon*).mp. [mp=title, abstract, original title, name of substance word, subject heading word, floating sub-heading word, keyword heading word, organism supplementary concept word, protocol supplementary concept word, rare disease supplementary concept word, unique identifier, synonyms] 757996<br>3 probiotic*.mp. [mp=title, abstract, original title, name of substance word, subject heading word, floating sub-heading word, keyword heading word, organism supplementary concept word, protocol supplementary concept word, rare disease supplementary concept word, unique identifier, synonyms] 35848<br>4 1 and 2 11345<br>5 3 and 4 56
**mbase <1974 to 2022 April 07>**	1 (systematic review* or meta-analys*).mp. [mp=title, abstract, heading word, drug trade name, original title, device manufacturer, drug manufacturer, device trade name, keyword heading word, floating subheading word, candidate term word] 609442<br>2 (dent* or tooth or teeth or orthodon* or oral surg* or endodon* or periodon* or prosthodon* or pedodon*).mp. [mp=title, abstract, heading word, drug trade name, original title, device manufacturer, drug manufacturer, device trade name, keyword heading word, floating subheading word, candidate term word] 714889<br>3 probiotic*.mp. [mp=title, abstract, heading word, drug trade name, original title, device manufacturer, drug manufacturer, device trade name, keyword heading word, floating subheading word, candidate term word] 53140<br>4 1 and 2 13267<br>5 3 and 4 98
**ISI Web of Science 131**	All fields=systematic review* OR meta-analys*<br>And All fields=dent* or tooth or teeth or orthodon* or oral surg* or endodon* or periodon* or prosthodon* or pedodon*<br>And All fields=probiotic*
**OPENGREY**<br>**0**	(dent* OR tooth OR teeth OR orthodon* OR "oral surg*" OR endodon* OR periodon* OR prosthodon* OR pedodon*) AND ("systematic review*" OR meta-analys*) AND (probiotic*)
**Sum: 202 out of 285 publications left after removing the duplicates**	

Table S2. Quality assessment.

trial	sponsorship	random	allocation	blinding1	blinding2	incomplete	selective
Alkaya 2016	0	0	0	0	0	0	0
Alshareef 2020	1	2	1	2	2	0	2
Ashwin 2015	1	0	0	0	0	0	1
Chandra 2016	1	0	1	0	0	0	1
Costacurta 2018	1	1	1	1	1	0	0
Dhaliwal 2017	0	0	1	0	0	0	0
Dugourd 2020	1	0	0	1	1	0	0
Elsadek 2020	0	1	1	0	0	0	1
Fawaz 2019	1	0	0	0	0	0	1
Flichy-Fernández 2015	1	0	0	0	0	0	0
Galofré 2018	0	0	0	0	0	0	0
Ghasemi 2017	1	1	1	0	1	0	0
Ghasemipour 2014	0	1	1	1	1	0	1
Hardar 2016	1	0	0	0	1	1	1
Ince 2015	0	0	0	0	0	0	0
Iniesta 2012	1	2	1	0	0	0	0
Invernici 2018	0	0	0	0	0	0	0
Iwasakia 2016	1	1	1	0	1	0	0
Jagadeesh 2017	1	2	0	0	0	0	0
Javid 2020	0	1	1	0	0	0	1
Keller 2012	1	2	0	0	0	0	0
Kuru 2017	0	0	0	0	0	0	0
Laleman 2015	1	0	0	0	0	0	0
Mani 2017	0	1	1	0	1	0	0
Marta 2019	0	0	0	0	0	0	0
Meenakshi 2018	1	2	0	1	2	0	2
Mimura 2017	1	0	0	1	1	1	0
Montero 2017	0	0	1	0	0	0	0
Morales 2016	0	0	0	0	0	1	0

Morales 2018	0	0	0	0	0	0	0
Nagarajappa 2015	1	1	2	0	1	0	1
Nozari 2015	0	1	1	0	1	0	1
Pedersen 2019	0	1	0	1	1	1	0
Pelekos 2019	0	0	0	0	0	0	0
Pena 2018	1	0	0	0	0	0	0
Penala 2016	0	0	0	0	0	1	0
Pinto 2014	1	0	2	2	0	2	0
Sajedinejad 2018	0	0	0	0	0	1	1
Srivastava 2016	1	0	0	1	0	0	1
Staab 2009	1	0	1	2	2	0	0
Stecksen-Blicks 2009	0	0	0	0	0	2	1
Suzuki 2014	0	0	0	0	0	1	1
Teanpaisan 2013	0	0	0	0	0	0	1
Tekce 2015	0	0	0	0	0	0	0
Teughels 2013	0	0	0	0	0	0	0
Toiviainen 2015	0	2	0	0	0	0	0
Vivekananda 2010	1	0	0	0	0	0	0
Yadav 2014	1	1	0	0	1	0	1

0=low 1=n=unclear 2=high
